# FliS/flagellin/FliW heterotrimer couples type III secretion and flagellin homeostasis

**DOI:** 10.1038/s41598-018-29884-8

**Published:** 2018-08-01

**Authors:** Florian Altegoer, Sampriti Mukherjee, Wieland Steinchen, Patricia Bedrunka, Uwe Linne, Daniel B. Kearns, Gert Bange

**Affiliations:** 1 LOEWE Center for Synthetic Microbiology & Dep. of Chemistry, Philipps University Marburg, Hans-Meerwein-Strasse 6, 35043 Marburg, Germany; 2 Department of Biology, Indiana University, 1001 East 3rd Street, Bloomington, IN 47405 USA

## Abstract

Flagellin is amongst the most abundant proteins in flagellated bacterial species and constitutes the major building block of the flagellar filament. The proteins FliW and FliS serve in the post-transcriptional control of flagellin and guide the protein to the flagellar type III secretion system (fT3SS), respectively. Here, we present the high-resolution structure of FliS/flagellin heterodimer and show that FliS and FliW bind to opposing interfaces located at the N- and C-termini of flagellin. The FliS/flagellin/FliW heterotrimer is able to interact with FlhA-C suggesting that FliW and FliS are released during flagellin export. After release, FliW and FliS are recycled to execute a new round of post-transcriptional regulation and targeting. Taken together, our study provides a mechanism explaining how FliW and FliS synchronize the production of flagellin with the capacity of the fT3SS to secrete flagellin.

## Introduction

The bacterial flagellum is among the most-studied bacterial nanomachines and represents one of the most powerful motors in the biosphere. Flagellar architecture is highly conserved among bacterial species and can be divided into: a membrane-embedded basal body including a flagella-specific type III secretion system (fT3SS), the cytoplasmic C-ring and the rod, extracellular hook and filament structures.

Flagella biogenesis follows a highly conserved sequence of assembly steps that is tightly regulated in time and space (reviewed in^1–4^). Dozens of assembly factors and chaperones orchestrate flagella assembly at the transcriptional, post-transcriptional, translational or post-translational level^5–8^. The most abundant flagellar building block is the flagellin protein that assembles in a helical pattern of more than 20,000 copies to form the extracellular filament^9^. Successful assembly of the flagellar filament relies on the FliS protein that prevents cytoplasmic aggregation of flagellin^10^ and guides flagellin to the cytoplasmic side of the fT3SS. At the fT3SS, the flagellin/FliS complex interacts with the cytoplasmic domain of the fT3SS transmembrane protein FlhA (FlhA-C)^11–13^. Therefore, FliS acts as targeting factor directing its flagellin client to the export gate of the fT3SS for subsequent secretion.

In a wide range of bacterial species, production of flagellin is post-transcriptionally regulated by the proteins CsrA and FliW^14–18^. In *Bacillus* *subtilis*, CsrA inhibits translation of flagellin by binding to two sites that are present within the 5′untranslated region (UTR) of the *hag* mRNA^14,19^. FliW allosterically antagonizes CsrA in a noncompetitive manner by excluding the 5′ UTR from the CsrA–RNA binding site, allowing the translation of flagellin^17,18^. FliW, which can also bind to flagellin, is sequestered as cytoplasmic levels of flagellin rise, allowing CsrA again to inhibit translation of the *hag* mRNA. This cycle is thought to enable homeostasis of cytoplasmic flagellin concentrations over a low and narrow threshold^20^.

While our knowledge on the functional roles of FliS, FliW and CsrA is steadily increasing, structural information is lagging behind. We present the high-resolution structure of FliS bound to full-length flagellin showing the FliS/Flagellin interaction is more complex than indicated by previous partial structures^21,22^. Moreover, we show that FliW and FliS bind to opposing interfaces located at the N- and C-termini of flagellin, respectively. The heterotrimeric complex of flagellin, FliS and FliW is competent to interact with FlhA-C, although FliW is not required for efficient interaction. This finding suggests to us that FliW remains sequestered until flagellin export by the fT3SS. After release, free FliW would bind and antagonize CsrA to enable further production of flagellin. Taken together, our data suggest a mechanism explaining how FliW and FliS might synchronize the production of flagellin with fT3SS secretion.

## Results

### Crystal structure of FliS in complex with full-length flagellin

We first wanted to determine a structure elucidating the complex of the FliS chaperone in complex with its client flagellin. Thus far, the only structural information that was available was of FliS bound to a C-terminal fragment of flagellin (residues 478–518; pdb: 1ORY,^21^, 4IWB,^22^). To obtain the complete structure, the flagellin/FliS complex from *B. subtilis* was co-expressed in *E*. *coli* BL21(DE3) and co-purified by nickel-ion affinity chromatography (IMAC) followed by size exclusion chromatography (SEC). Multi-angle light scattering analysis indicated that the *B*. *subtilis* flagellin/FliS complex forms a heterodimer (Fig. S1a).

**Figure 1:**
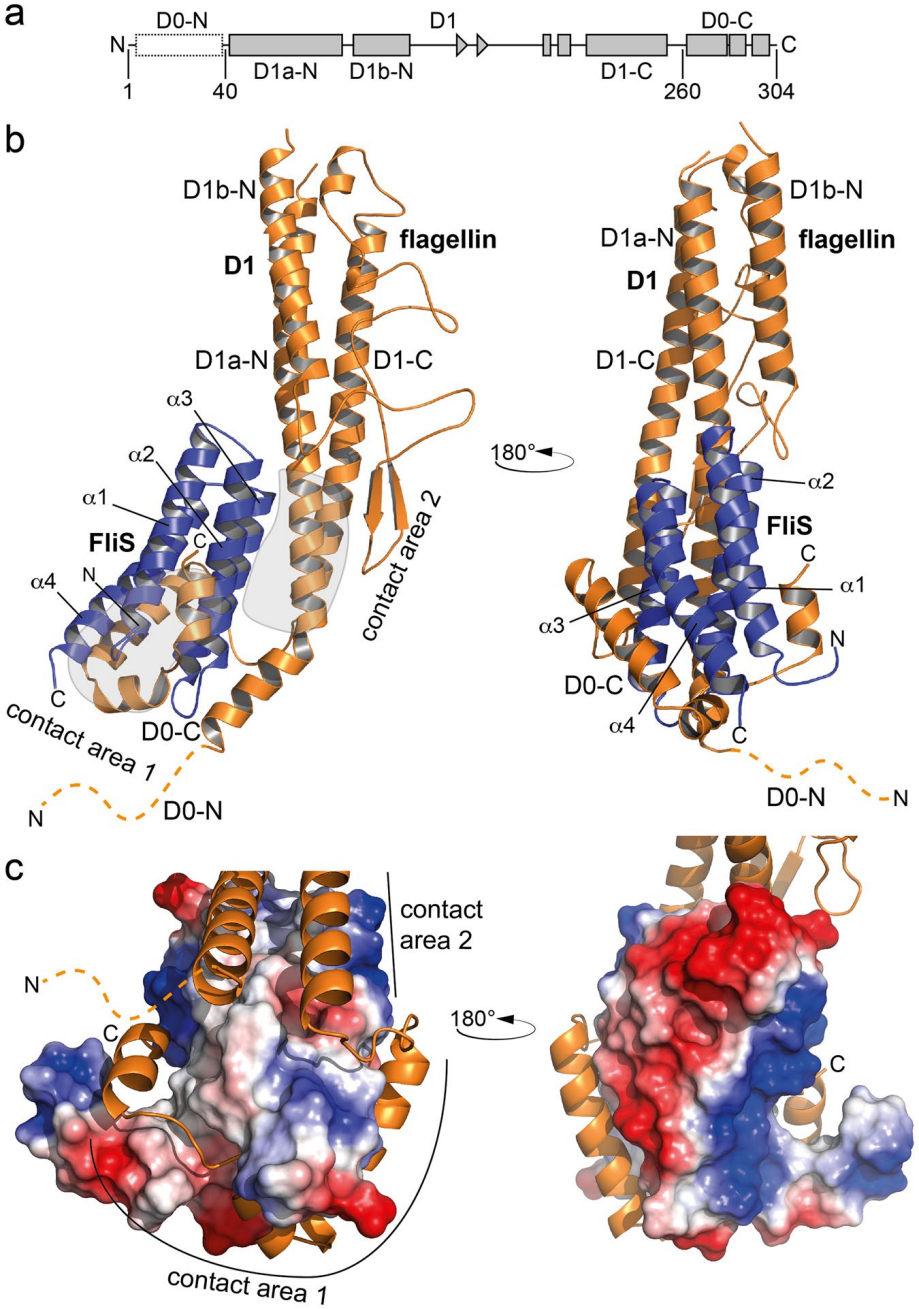
Crystal structure of the flagellin/FliS heterodimer. (**a**) Domain organization of flagellin. (**b**) Crystal structure of flagellin/FliS as a cartoon representation in two orientations. flagellin and FliS are shown in orange and blue, respectively. ‘N’ and ‘C’ indicate N- and C-termini, respectively. The disordered N-terminus of flagellin is shown as dashed line. Contact areas between flagellin and FliS are encircled in grey. (**c**) Electrostatic surface potential of FliS within the contact area of flagellin to FliS. The flagellin-binding pocket is of hydrophobic and polar nature (*left side*), whereas the opposite site of FliS is highly charged (*right side*).

Crystals of the flagellin/FliS complex were obtained in two spacegroups, the primitive monoclinic space group *P*2_1_ at 1.5 Å resolution and the primitive orthorhombic spacegroup *P*22_1_2_1_ at 2.1 Å resolution. The flagellin/FliS heterodimer shows overall dimensions of 95 Å, 40 Å and 40 Å (Fig. 1b, Table S1) and could be built to completeness except for the first 45 highly disordered residues at the N-terminus of flagellin (Fig. 1a,b, dashed line).

The structure of *B*. *subtilis* flagellin is similar to the flagellin of other bacterial species^23^ and can be divided into D0-N, D1 and D0-C domains (Fig. 1a, S1b). The crystal structure suggests that the flagellin/FliS complex forms a rather compact particle detectable also in solution by small angle X-ray scattering (SAXS). The two proteins interact by an extensive network of hydrophobic and electrostatic interactions with an interface area of 2600 Å^2^ which can be divided into contact areas 1, 2 and 3.

Contact area 1 involves the C-terminus of flagellin (D0-C) wrapped around the surface of FliS in an extended horseshoe like conformation with a mainly hydrophobic interaction area of 1600 Å (Contact area 1; Fig. 1b,c). Contact area 1 is highly similar to that in an earlier structure of FliS bound to the C-terminus of flagellin^21^ with a Cα r.m.s.d of 2.2 Å over 140 amino acid residues (Fig. S1c). Contact area 2 aligns FliS to the D1 core domain of flagellin, a region that was not present in previous crystal structures^24–26^. Interactions within this interface are of more electrostatic nature (Contact area 2, Fig. 1b,c). Notably, residues 46 to 57 are kinked by 45° in the orthorhombic structure, while the helix is straight in the crystal structure derived from monoclinic crystals (Fig. S2). Apart from this difference, both structure superimpose well with a r.m.s.d of 1.5 over 329 Cα-atoms.

Taken together, our crystal structure shows that the interaction between flagellin and FliS involves a complex interface at flagellin including the D1 and D0C domains.

### Flagellin/FliS and FlhA-C interact via a complex interface

**Figure 2:**
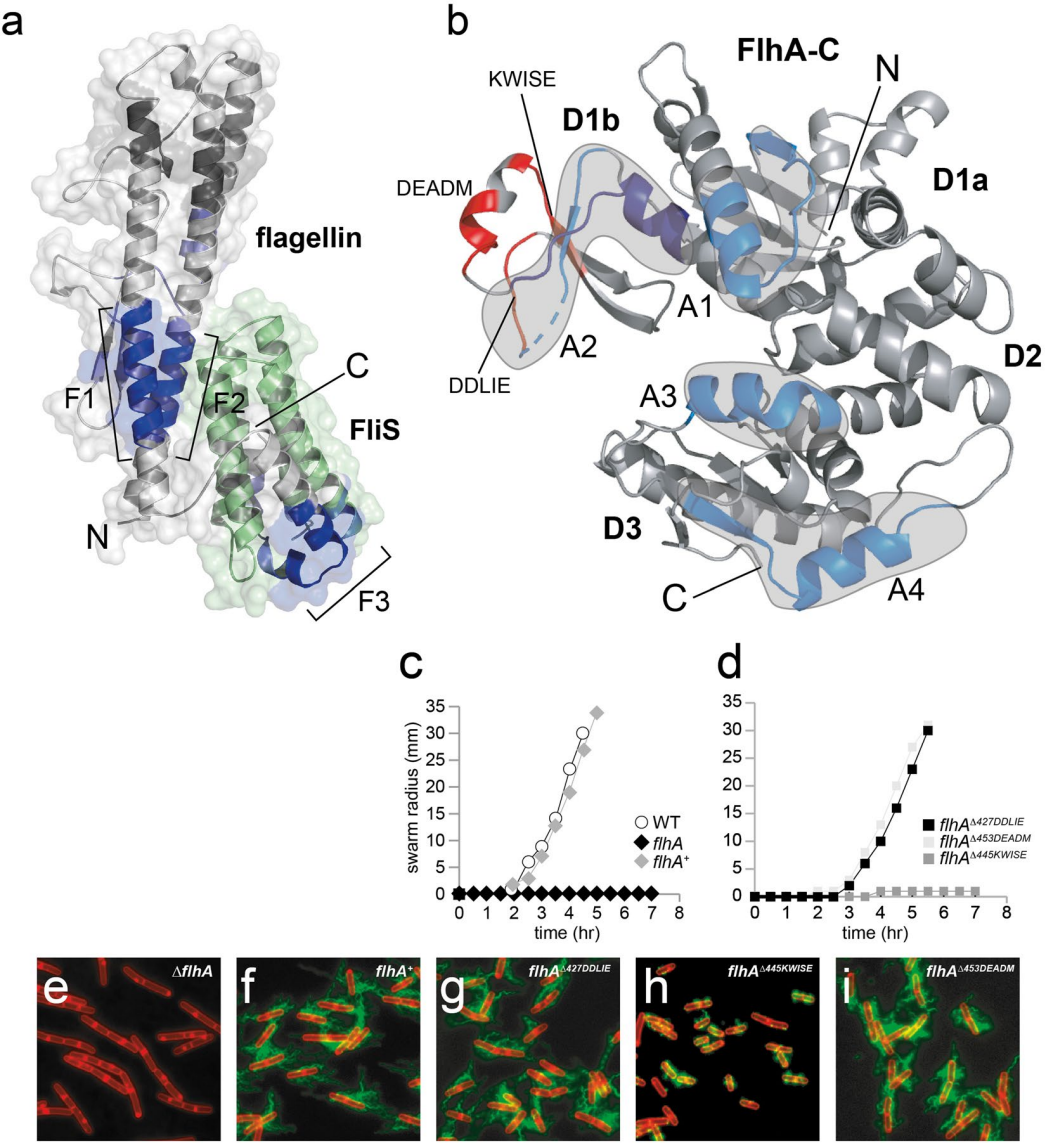
Recruitment of the FliS/flagellin/FliW complex to FlhA-C. (**a**) HDX analysis of flagellin/FliS in the presence of FlhA-C. Difference in HDX-labelling is shown in different shades of blue (dark blue: strong protection; light blue: weak protection) in Dalton. Regions in the cartoon representation of flagellin/FliS that exchange less in the presence of FlhA-C are highlighted (F1, F2 and F3). FliS is shown in green as no HDX-data were obtained. (**b**) HDX analysis of FlhA-C in the presence of flagellin/FliS. The difference in HDX-labelling is shown. Blue regions in the cartoon representation of FlhA-C exchange less in the presence of flagellin/FliS and are encircled in grey (A1, A2, A3 and A4). Peptides deleted within the D1b domain are highlighted in red. (**c**,**d**) Quantitative swarm expansion assay for *flhA* mutant and complementation strains: WT is swarming proficient and covers a 0.7% LB agar plate in about 4.5 hours but Δ*flhA* is non-swarming. The *flhA*^*Δ445KWISE*^ mutant strain shows also a strong swarming deficiency (**e**–**i**) Fluorescence microscopy of *B*. *subtilis* showing deficiency in filament assembly of a *flhA* and *flhA*^*Δ445KWISE*^ mutant. Scale bars are 2 µm.

With the structure of full-length flagellin bound to FliS in hand, we wanted to understand how flagellin/FliS interacts with FlhA-C. Hence, we performed hydrogen-deuterium exchange (HDX) mass spectrometry (HDX-MS). This method allows monitoring conformational changes within proteins and the determination of interaction sites^27^. FlhA-C, flagellin/FliS and the FlhA-C/flagellin/FliS complex were incubated in deuterated buffer for different times, digested with pepsin and the resulting peptic fragments analyzed by electrospray ionization-mass-spectrometry (Fig. S3c,d). When bound to FlhA-C, a significant decrease in HDX of flagellin/FliS was detected in three regions of flagellin at D1-N, D1-C and D0-C (termed: F1-F3). The region within D1-N includes residues 61–72 (Fig. 2a,F1), but slight protection is also visible from residue 123–142. The most prominent HDX protection can be observed in the D1/D0-C of flagellin ranging from residues 236–249 and 275–300 (Figs 2a,F2,F3, S3c). While the first patch is within the D1-C domain of flagellin, residues of the latter are arranged in two short α-helices connected by a loop and wrap around FliS (Fig. 2a).

We now inspected regions at FlhA-C that become protected from HDX upon binding of flagellin/FliS. In total, the binding interface covers four regions (termed: A1-A4). Firstly, a small patch ranging from residues 371–390 within the D1a domain shows protection (Figs 2b,A1, S3d). Further residues within the D1b domain (416–428 and 460–471) exchange less in the presence of flagellin/FliS (Figs 2b,A2, S3d). Lastly, residues 562–571 (A3) and 579–596 (A4) show a dramatic decrease in deuteron incorporation, indicating stabilization or shielding (Figs 2b, S3d). These results show that the D1b domain and adjacent regions within the D3 provide the major interaction site for the flagellin/FliS complex on FlhA-C.

To substantiate our *in vitro* findings for the flagellin/FliS interaction with FlhA-C and their implications on motility and the flagellation state of *B*. *subtilis*, we tested the consequence of deleting *flhA* as well as several patches at FlhA on swarming motility and flagellin secretion (Fig. 2c–i).

Whereas wildtype cells swarmed rapidly atop the surface of an agar Petri plate, cells mutated for *flhA* exhibited a severe defect in filament assembly and motility. The phenotype was rescued when *flhA* was expressed from the native *flhA* promoter inserted at an ectopic site in the chromosome (*amyE::P*_*flhA*_*-flhA*) (Fig. 2c). Next, three “five amino-acid” deletions in the *flhA* open reading frame (*flhA*^*Δ427DDLIE*^, *flhA*^*Δ445KWISE*^, *flhA*^*Δ453DEADM*^) were expressed from the *P*_*fla/che*_ promoter inserted at an ectopic locus. We found that the *flhA*^*Δ445KWISE*^ mutant was impaired for filament assembly and swarming motility *in vivo* suggesting that this patch of residues is required for interaction between FlhA-C and flagellin (Fig. 2d,h). This peptide is part of the D1b domain at FlhA-C and in close proximity to region A2 (Fig. 2b). In fact, the amino acids KWIS are part of a β-sheet that stabilizes the D1b domain and represents an interaction interface of the flagellin/FliS complex as shown by our HDX-data.

**Figure 3:**
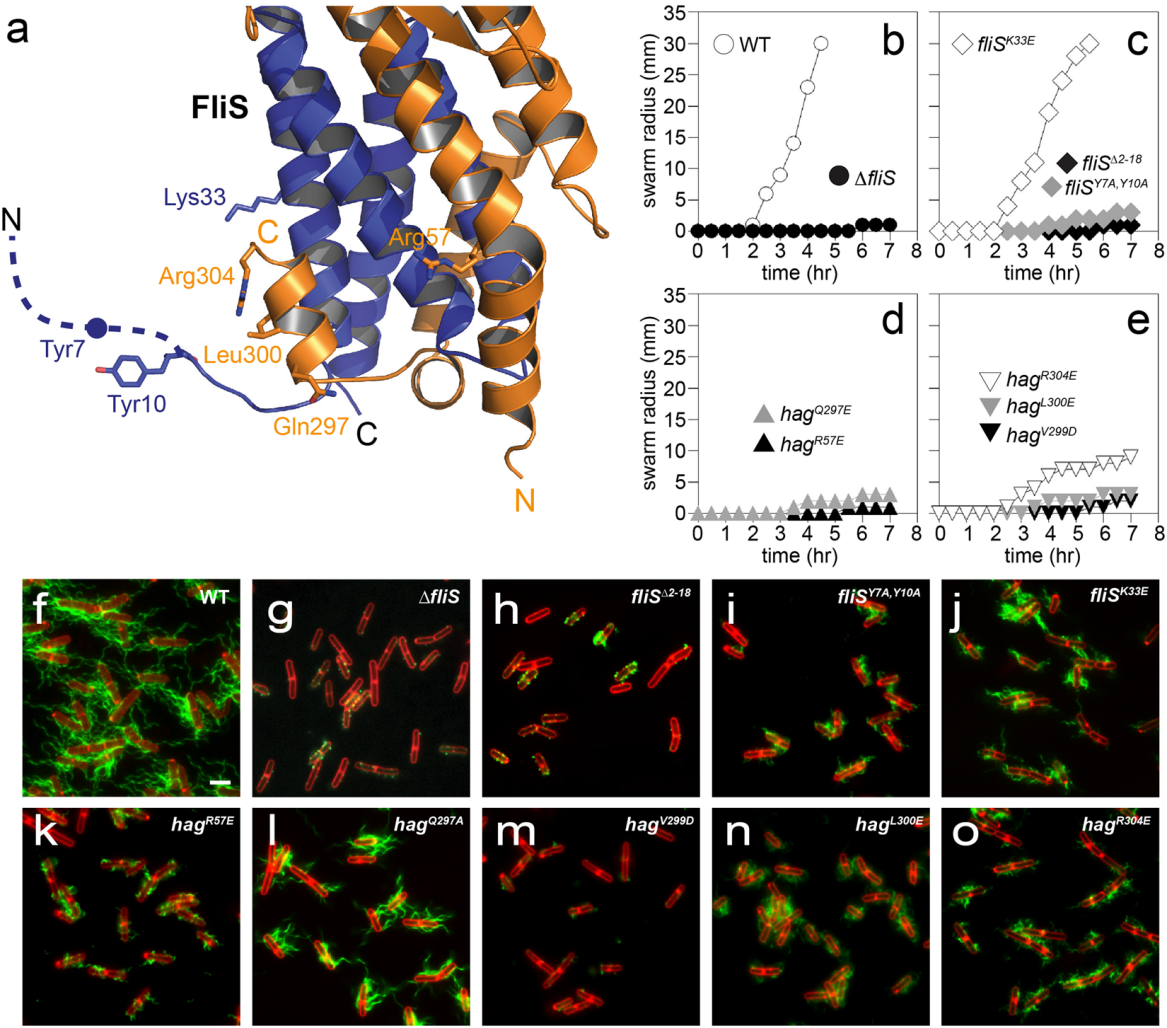
Physiology of flagellin/FliS binding to FlhA-C. (**a**) Projection of different amino acid exchanges onto the structure of flagellin/FliS. Flagellin is shown in orange, while FliS is depicted in blue. Dashed lines indicate disordered regions. (**b**–**e**) Quantitative swarm expansion assay for *fliS* and *flagellin* (*hag*) mutants: WT is swarming proficient and covers a 0.7% LB agar plate in about 4.5 hours but Δ*fliS* is non-swarming. Both *fliS*^*Δ2–18*^ and *fliS*^*Y7A*,*Y10A*^ phenocopy a ΔfliS mutant for swarming. *fliS*^*K33E*^ is swarming proficient. *hag*^*R57E*^ confers a non-swarming phenotype while all *hag* mutants except *hag*^*V299D*^ show almost no swarming motility. (**f**–**o**) Fluorescence microscopy of *B*. *subtilis* filaments shows deficiency of Δ*fliS*, *fliS*^*Δ2-18*^ and *fliS*^*Y7A*,*Y10A*^ mutants to secrete flagellin. *fliS*^*K33E*^ shows short flagellar filaments. *hag*^*R57E*^, *hag*^*V299D*^, *hag*^*L300E*^ and *hag*^*R304E*^ show almost no flagellar filaments, while *hag*^*Q297A*^ exhibits short flagellar filaments. Scale bars are 2 µm.

In addition, we generated several mutants in the binding interface of flagellin and FliS at FlhA (Fig. 3). The N-terminus of FliS has earlier been described to be important for the recognition by FlhA-C in *S*. *typhimurium*. More precisely, Tyr10 at FliS has been shown to be critical for interaction of FliC/FliS with FlhA *in vitro* and Salmonella strains with a variation of Tyr10 showed drastically reduced motility^5^.

Indeed, FliS variant lacking the first 18 residues phenocopied the *fliS* deletion strain in that it was both strongly impaired for filament formation and swarming motility (Fig. 3b,c,g,h). Variation of tyrosine 7 and 10 at FliS to alanine also reflected this phenotype (Fig. 3c,i), supporting the importance of these two residues for FlhA-C recognition as they are not involved in the interface of flagellin at FliS (Fig. 3a). The variation of lysine 33 to glutamate, however, showed no severe motility defect but nonetheless reduced the amounts of secreted flagellin (Fig. 3c,j). Finally, we varied amino acid residues of flagellin, which are on opposing sites of the interaction interface with FliS (Fig. 3a). While some of the variations (e.g. Q297A and R304E) still showed a partial flagellation (Fig. 3l,o), swarming motility was almost completely impaired (Fig. 3d,e). Each of the mutant alleles of flagellin were as stable as the wild type in the cytoplasm supporting the interpretation that their defect was likely in secretion. (Fig. S4a). Taken together, the *in vivo* data reflect our HDX experiments by confirming that residues within the patches F2 and F3 (Figs 2a,F2,F3, S3c) at flagellin are important for the recognition of the flagellin/FliS complex at FlhA-C.

### Interaction of FliW with the flagellin/FliS complex

**Figure 4:**
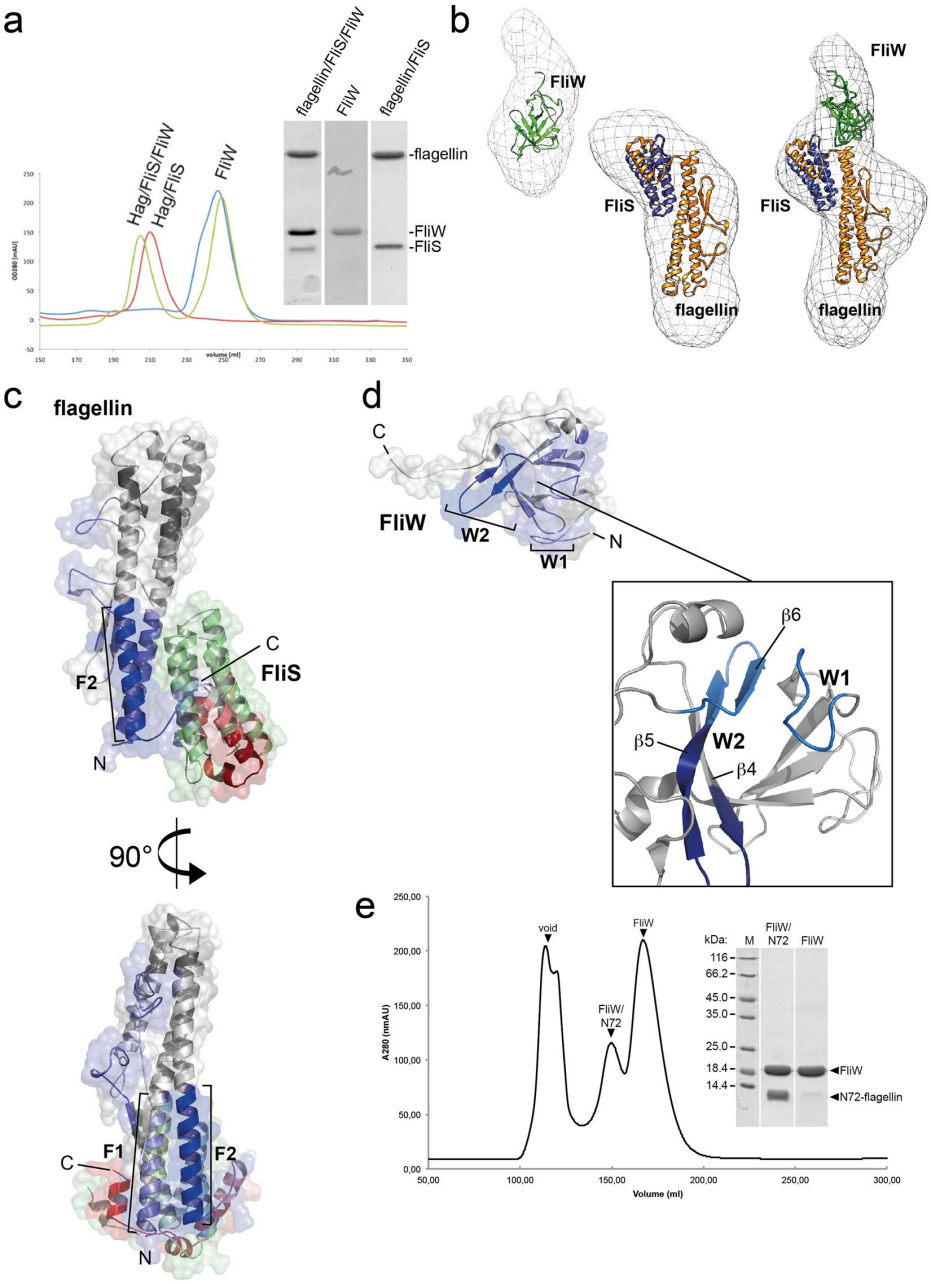
SAXS- and HDX-analysis of the FliS/flagellin/FliW interaction. (**a**) Size-exclusion chromatograms (SEC) of flagellin/FliS (red), FliW (blue) and FliS/flagellin/FliW (green). The inset shows a Coomasse-stained SDS PAGE of the peak fractions. (**b**) SAXS analysis of FliW, flagellin/FliS and FliS/flagellin/FliW. The crystal structures have been fitted to the SAXS-density with the docking algorithm implemented in Chimera. (**c**) HDX analysis of flagellin/FliS versus flagellin/FliW. The difference in HDX-labelling is shown from blue (less exchange) to red (more exchange) in Dalton. Blue regions in the cartoon representation of flagellin/FliS exchange less in the presence of FliW (F1 and F2), whereas red regions get unprotected. (**d**) HDX analysis of FliW versus flagellin/FliW. The difference in HDX-labelling is shown from blue (less exchange) to red (more exchange) in Dalton. Blue regions in the cartoon representation of FliW exchange less in the presence of flagellin (W1 and W2). (**e**) SEC-chromatogram of a flagellin_N72_/FliW complex (*left*) and a Coomassie-stained SDS-PAGE of the two peak fractions (*right*).

Next, we wanted to understand the interaction of FliW with flagellin on the flagellin/FliS complex at the molecular level. Co-purification of FliW, FliS and flagellin yielded a stable heterotrimer on SEC (Fig. 4a). However, all attempts to crystallize the complex were unsuccessful. To determine where FliW bound to the flagellin/fliS complex, we employed SAXS. Comparing the SAXS shape of flagellin/FliS (see above) with that of the FliS/flagellin/FliW complex shows an extra density in close proximity to the N-terminus of flagellin and opposite of the FliS binding site at flagellin (Fig. 4b). These data show that FliW interacts with a region close to the N-terminus of flagellin.

To further specify the interaction site of FliW at flagellin and *vice versa*, we performed HDX-MS. Therefore, FliW alone and in complex with flagellin was incubated in deuterated buffer for different times, digested with pepsin and the resulting peptic fragments analyzed by electrospray ionization-mass-spectrometry. Similar to flagellin/FliS, flagellin and FliW form a stable complex on SEC (Fig. S4b). HDX-MS indicated that FliW protected two adjacent regions at flagellin spanning the N-terminal part (Figs 4c, S3a; i.e. residues 11–34, 50–72) of helix D1a-N (region F1) and the C-terminal part (Figs 4c, S3a, residues 240–260) of helix D1-C (region F2). Conversely, flagellin protected a loop region close to the N-terminus of FliW (Figs 4d, S3b, residues 3–11, W1) and the three β-strands β4, β5, and β6 (Figs 4d, S3b, residues 22–28, 95–115; W2). Thus, our HDX-MS and SAXS experiments supported one another as both indicated that FliW interacts with the N-terminal fraction of flagellin.

To determine whether the N-terminus of flagellin was sufficient for FliW interaction, we generated several N-terminal flagellin constructs, with varying length and tested them for FliW binding. Only two constructs, namely N60-flagellin (containing the N-terminal 60 residues of flagellin) and N72-flagellin were soluble and capable of FliW binding. Due to the higher stability on SEC, we co-purified N72-flagellin with FliW as described above and reconstituted the complex on SEC (Fig. 4e). Hence, we can show that the N-terminus of flagellin is sufficient for recognition by FliW. Taken together, our experiments show that FliW and FliS bind to opposing sites at flagellin (Fig. 4b,c).

### The FliS/flagellin/FliW complex interacts with FlhA

**Figure 5:**
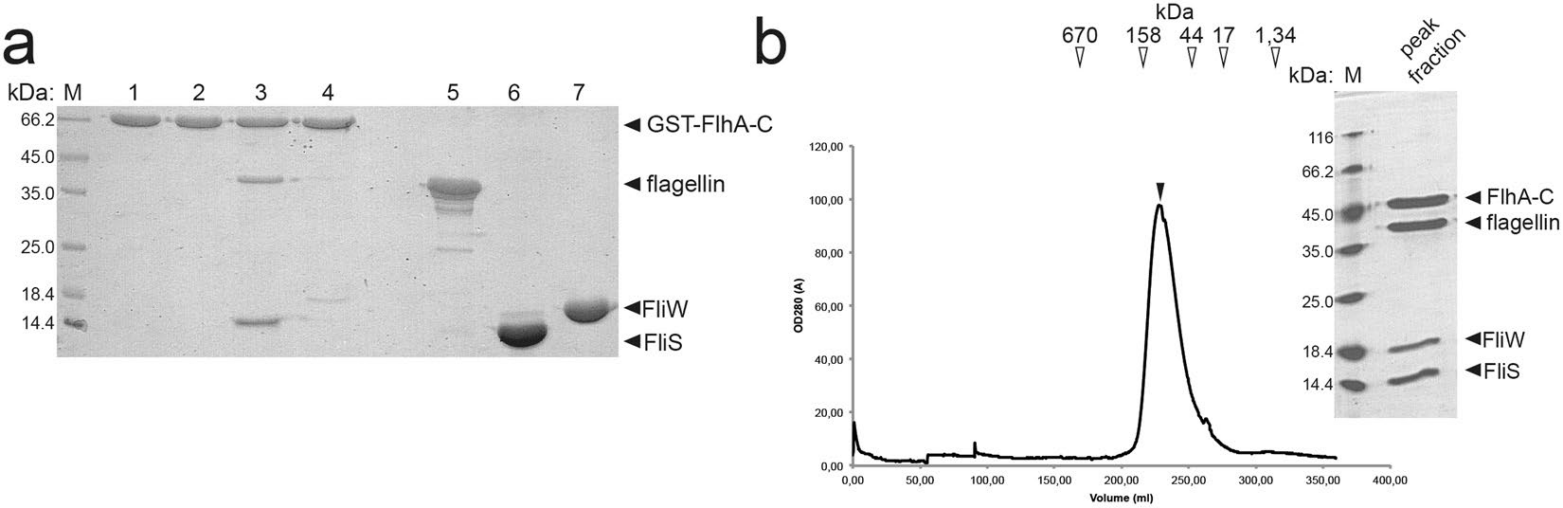
FliW is not a prerequisite for flagellin recognition by FlhA-C. (**a**) Coomassie-stained SDS-PAGE of a pulldown assay employing GST-tagged FlhA-C. Flagellin alone does not interacts with FlhA-C (lane 2). FliS alone is able to recruit flagellin to FlhA-C (lane 3), while FliW alone is not (lane 4). Lanes 1, 5, 6 and 7 represent the input of purified GST-FlhA-C, Flagellin, FliS and FliW, respectively. (**b**) Reconstitution of the FliS/flagellin/FliW/FlhA-C complex on size exclusion chromatography shows an apparent heterotetramer. Arrows indicate the protein size standard in kDa. The inset shows a Coomassie-stained SDS-PAGE of the peak fraction.

FliS is required for the recruitment of flagellin to the cytoplasmic domain of the fT3SS transmembrane protein FlhA (FlhA-C)^11,28,29^. However, whether FliW would influence binding of flagellin/FliS to FlhA-C was unknown. Whereas FliS recruited flagellin to FlhA-C, FliW did not (Fig. 5a). These findings suggest that FliW is not required for recruitment of flagellin to FlhA-C. However, it does not exclude that FliW could still be bound to a flagellin/FliS complex interacting with FlhA-C. To investigate such a scenario, we reconstituted a heterotetrameric complex consisting of FliS/flagellin/FliW and FlhA-C on SEC. This experiment shows at the biochemical level that the FliS/flagellin/FliW heterotrimer can interact with FlhA-C (Fig. 5b). Our experiment also suggests that FliS and not FliW is key to recognition of flagellin at FlhA. Taken together, FliS but not FliW is required for flagellin recognition at FlhA-C, although FliW might still be bound to flagellin at this point.

## Discussion

The production of flagellin represents a critical step during flagellar assembly and a prominent example of a multi-layer regulation. Once the hook-basal-body complex is completed, secretion of the anti-σ-factor FlgM enables the σD dependent transcription of late stage genes including the *hag* gene encoding flagellin^30–32^.

**Figure 6:**
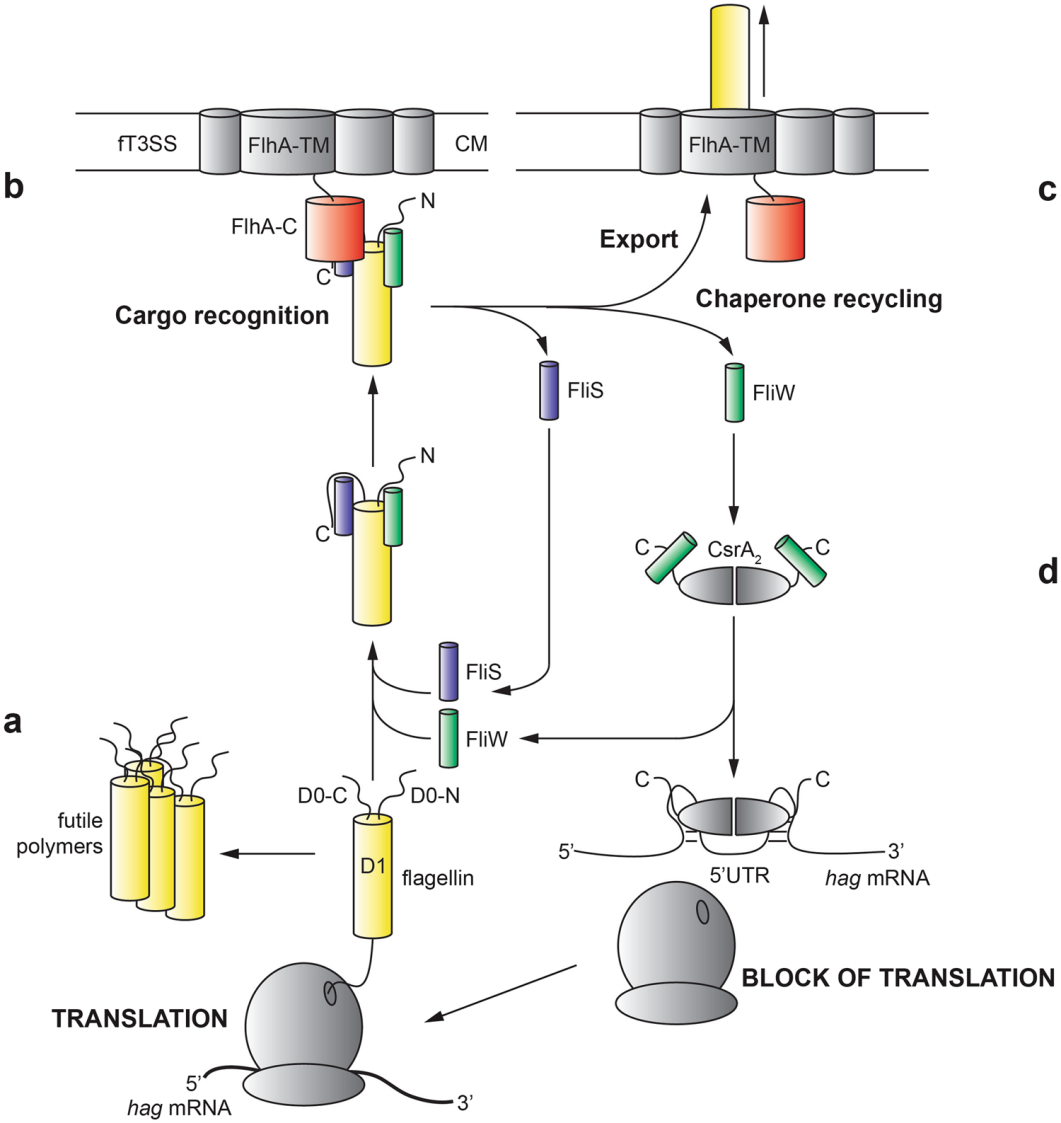
Model for coupling homeostasis and type-III-secretion of flagellin. The figure depicts how FliS, FliW and CsrA act in concert to couple flagellin homeostasis with its export by the fT3SS. Flagellin is shown in yellow, FliW in green, FliS in blue and FlhA-C in red. CsrA was not part of this study and is shown in grey. (**a**) FliS and FliW bind to flagellin once it has emerged from the ribosome to prevent formation of futile aggregates. (**b**) The FliS/flagellin/FliW complex is recognized by the type III secretion system via FlhA-C. (**c**) FliS and FliW are recycled after flagellin secretion. (**d**) Free FliW can sequester CsrA from the *hag* leader transcript, thereby allowing the next round of flagellin translation.

Once flagellin translation is initiated and flagellin nascent chains emerge from the ribosome, they are recognized by FliW and FliS, which bind to the D0-N, and the D1-N and D0-C domains of flagellin, respectively (Fig. 6a). Simultaneous binding of FliS and FliW has two effects on the production pathway of flagellin: (i) FliS binding to the C-terminus ensures that flagellin must be fully translated to be primed for export. Moreover, formation of futile flagellin aggregates is prevented by the sequestration of the C-terminus by FliS. FliS wraps around the D0-C domain and interacts with flagellin via a complex network also employing part of the D1-C domain (Fig. 1b,c). (ii) FliW binds to two regions at flagellin (the N-terminal part of the D1N-domain and a region within the D1C-domain (Fig. 4c). This interaction sequesters FliW and allows CsrA to block the next round of flagellin translation. By this mechanism, only a limited number of flagellin molecules are produced simultaneously and subsequently secreted. The interaction of CsrA and FliW has been elucidated at atomic resolution and the regulation and interaction of CsrA with the *hag* mRNA is well-understood^17–19^. The biochemical data obtained in this study show that the N-terminal 72 residues of flagellin are sufficient for FliW binding (Fig. 4e). However, a second binding site of FliW at flagellin in the D1-C region shows a prominent protection upon HDX (Figs 4c,F2, S3a) demonstrating that FliW also binds to the D1-C at flagellin as shown previously^33^.

The heterotrimeric complex of FliS/flagellin/FliW is then recognized by FlhA-C within the fT3SS (Fig. 6b). We could show that recognition of flagellin at FlhA-C is solely dependent on FliS, and bound-FliW does not disturb this process (Fig. 5a,b). As there is no evidence that FliW is recycled prior to binding of the FliS/flagellin/FliW complex to FlhA-C and the chaperone recycling process is poorly understood, FliW might stay attached to flagellin until its client is secreted. However, precisely when FliW is released, either prior to the intiation of flagellin secretion or concomitant with export cannot be distinguished by these data.

FlhA provides an entry gate to all proteins that are secreted via the fT3SS^5,11,12,34^. Our findings in *B*. *subtilis* confirm the results from *S*. *typhimurium*^5^ that the flagellin/FliS complex binds to residues at FlhA-C within the groove between the D1b and D3 domain (Fig. 2b). Furthermore, the recent co-structure of FlhA-C with a FliS:FliC fusion allowed insights into how the N-terminal helix of FliS penetrates into the groove in close proximity to the D1b, only in the flagellin-bound state^35^. These data also confirm our HDX results that indicate a strong protection within the D1b and adjacent regions of the D1a domain of FlhA-C upon binding of flagellin/FliS (regions A1 and A2; Fig. 2b) and explain the importance of the N-terminus of FliS for flagellin secretion (compare Fig. 3c,h).

In contrast to the co-structure by Xing and coworkers^35^, the data obtained in this study indicate a direct interface of flagellin at FlhA-C covering not only the D0-C but also residues within the D1-C and the D1-N (Figs 2a, S3c). A full-length flagellin/FliS/FlhA-C structure might be necessary to understand the interaction in more detail. These residues are also part of the FliS and FliW interface at flagellin (compare Fig. 4c). A possible explanation of overlapping binding sites of FlhA-C and FliS/FliW at flagellin might be that the two proteins are part of a quality control step prior to flagellin secretion. These overlapping binding sites might also be part of the mechanism that allows the release of FliW and FliS prior to the release of flagellin into the pore of the fT3SS.

Once flagellin has been recognized by FlhA-C, flagellin should be secreted by the fT3SS (Fig. 6c). Translocation of flagellin across the plasma membrane leads to release of FliS and FliW, which in turn would be free to initiate a new round of translation and guiding the nascent flagellin molecule towards FlhA-C at the cytoplasmic base of the fT3SS (Fig. 6d).

The mechanism of how flagellin is recognized by FliS shares certain similarities with the recognition of the needle protein SctF in enteric pathogens such as e.g. *Yersinia*, *Shigella* and *Pseudomonas* species. SctF is the major constituent of the injectisome needle, a structure paralogous to hook and filament structures in the bacterial flagellum, although the degree of conservation is low^1,36^. Briefly, secretion of the needle protein requires the presence of two chaperones, PscE and PscG (*Pseudomonas* nomenclature used for simplicity) that sequester the amphipathic C-terminus of SctF^37,38^. The interaction between SctF and PscG is mainly mediated by hydrophobic interactions and therefore reminiscent of the flagellin/FliS interaction within contact area 1 (compare Fig. 1b), despite of structural differences between the two complexes. In both cases, an abundant protein with the intrinsic property to aggregate is stabilized by the help of a chaperone protein.

The protein PscE however, has been shown to stabilize the first chaperone PscG *in vitro*^39^ and is therefore also an essential component of the heterotrimeric complex SctF-PscG-PscE. In our case, FliW can stabilize flagellin *in vitro* (Fig. S4) but a FliS/FliW interaction has not been observed to date. Instead, FliW is part of the post-transcriptional regulation by CsrA, a mechanism that has not been shown to control SctF homeostasis and secretion. Based on the available data, one can only speculate whether the FliS/flagellin/FliW and the SctF-PscG-PscE complexes might share more similarities than anticipated so far. Taken together, the structural and functional differences between flagellin and SctF might have led to specific needs within the flagellum and the injectisome.

## Materials and Methods

### Protein production and purification

Protein production and purification was performed as described earlier^40^. More information can be found in the Supp. Mat. section.

### Crystallization and Structure Determination

Crystallization was performed by the sitting-drop method at 20 °C in 0.6 µl drops consisting of equal parts of protein and precipitation solutions. Flagellin/FliS crystallized at 50 mg/ml concentration within 1 to 5 days in 0.2 M Lithium sulfate, 0.1 M Tris pH 7.0 and 2.0 M Ammonium sulfate and 0.1 M Na-Cacodylate pH 6.5, 5% PEG 8000, 40% MPD. Prior data collection, crystals were flash-frozen in liquid nitrogen employing a cryo-solution that consisted of mother-liquor supplemented with 20% glycerol. Data were collected under cryogenic conditions at the European Synchrotron Radiation Facility at beamline ID29.

Data were integrated and scaled with XDS^41^ and merged with ccp4-implemented AIMLESS^42^. Structures were determined by molecular replacement with PHASER^43^, manually built in COOT^44^, and refined with PHENIX^45^. Structures of the flagellin/FliS complex were determined by molecular replacement using the crystal structures of the *S*. *typhimurium* flagellin core (pdb: 1IO1) and *B*. *subtilis* FliS (pdb code: 1VH6) as search models. Figures were prepared with Pymol^46^ and Chimera^47^.

### *In vitro* pull-down assays

For *in vitro* pull-down assays all proteins were produced in *E*. *coli* BL21 (DE3) and purified as described earlier^40^. Glutathione-s-transferase (GST)-tagged protein was used as bait and pre-incubated with glutathione (GSH) sepharose in a buffer containing 20 mM HEPES-Na (pH 7.5), 200 mM NaCl, 20 mM KCl, and 20 mM MgCl_2_. Excess of protein was removed by centrifugation and the GST-tagged protein immobilized on the sepharose incubated with different proteins. The sepharose beads were washed three times with buffer and finally eluted in 20 mM HEPES-Na (pH 7.5), 200 mM NaCl, 20 mM KCl, and 20 mM MgCl_2_, 50 mM reduced GSH. Elutes were analyzed by Coomassie-stained SDS-PAGE.

### Hydrogen-deuterium exchange mass-spectrometry (HDX-MS)

To analyze protein-protein interfaces by HDX-MS, proteins were incubated without or in the presence of the respective binding partners prior to H/D exchange. The mixtures were diluted in D_2_O-containing SEC to start the H/D exchange and the reaction stopped at different time points. Peptic peptides were generated by an online pepsin column and separated by reversed-phase HPLC. Data were analyzed using PLGS and DynamX 3.0 (Waters).

### Strain construction

All constructs were first introduced into the domesticated strain PY79 by natural competence and then transferred to the undomesticated 3610 background using SPP1-mediated generalized phage transduction^48,49^. All strains used in this study are listed in Table S2. All primers used in this study are listed in Table S3.

### SPP1 phage transduction

To 0.2 ml of dense culture grown in TY broth (LB broth supplemented after autoclaving with 10 mM MgSO_4_ and 100 µM MnSO_4_), serial dilutions of SPP1 phage stock were added and statically incubated for 15 min at 37 °C. To each mixture, 3 ml TYSA (molten TY supplemented with 0.5% agar) was added, poured atop fresh TY plates, and incubated at 37 °C overnight. Top agar from the plate containing near confluent plaques was harvested by scraping into a 50 ml conical tube, vortexed, and centrifuged at 5,000 × g for 10 min. The supernatant was treated with 25 µg/ml DNase final concentration before being passed through a 0.45 µm syringe filter and stored at 4 °C.

Recipient cells were grown to stationary phase in 2 ml TY broth at 37 °C. 0.9 ml cells were mixed with 5 µl of SPP1 donor phage stock. Nine ml of TY broth was added to the mixture and allowed to stand at 37 °C for 30 min. The transduction mixture was then centrifuged at 5,000 × g for 10 min, the supernatant was discarded and the pellet was resuspended in the remaining volume. Cell suspension (100 µl) was then plated on TY fortified with 1.5% agar, the appropriate antibiotic, and 10 mM sodium citrate.

### Microscopy

Fluorescence microscopy was performed with a Nikon 80i microscope with a phase contrast objective Nikon Plan Apo 100X and an Excite 120 metal halide lamp. FM4–64 was visualized with a C-FL HYQ Texas Red Filter Cube (excitation filter 532–587 nm, barrier filter >590 nm).

For fluorescent microscopy of flagella, 0.5 ml of broth culture was harvested at 0.5–2.0 OD_600_, and washed once in 1.0 ml of PBS buffer (137 mM NaCl, 2.7 mM KCl, 10 mM Na_2_HPO_4_, and 2 mM KH_2_PO_4_). The suspension was pelleted, resuspended in 50 µl of PBS buffer containing 5 µg/ml Alexa Fluor 488 C_5_ maleimide (Molecular Probes), and incubated for 5 min at room temperature^50^. Cells were then washed twice with 500 µl PBS buffer. When appropriate, membranes were stained by resuspension in 50 µl of PBS buffer containing 5 µg/ml FM4–64 (Molecular Probes) and incubated for 10 min at room temperature. Three microliters of suspension were placed on a microscope slide and immobilized with a poly-L-lysine-treated coverslip.

### Swarm expansion assay

Cells were grown to mid-log phase at 37 °C in LB broth and resuspended to 10 OD_600_ in pH 8.0 PBS buffer (137 mM NaCl, 2.7 mM KCl, 10 mM Na_2_HPO_4_, and 2 mM KH_2_PO_4_) containing 0.5% India ink (Higgins). Freshly prepared LB containing 0.7% Bacto agar (25 ml/plate) was dried for 20 min in a laminar flow hood, centrally inoculated with 10 µl of the cell suspension, dried for another 10 min, and incubated at 37 °C. The India ink demarks the origin of the colony and the swarm radius was measured relative to the origin. For consistency, an axis was drawn on the back of the plate and swarm radii measurements were taken along this transect. For experiments including IPTG, cells were propagated in broth in the presence of IPTG, and IPTG was included in the swarm agar plates.

### Significance statement

The ability to move towards favorable and avoid unfavorable conditions is key to the survival of many bacterial species. Bacterial movement relies on a sophisticated nanomachine, the flagellum. The major constituent of the flagellar filament is the protein flagellin that assembles into a helical filament with more than 20,000 monomers. The high abundance of this protein requires a sophisticated regulatory mechanism ensuring a tight coupling between production and secretion of flagellin. Here, we present the molecular framework of how flagellin homeostasis is coupled to its export by the proteins FliS and FliW.

### Data availability

Atomic coordinates and structure factors of flagellin/FliS have been deposited in the Protein Data Bank (PDB) with the accession codes 5MAW and 6GOW.

## Electronic supplementary material

Supporting Information
