# Supplementary material for: FliS/flagellin/FliW heterotrimer couples type III secretion and flagellin homeostasis
Source: Sci Rep. 2018 Aug 1;8:11552. doi: 10.1038/s41598-018-29884-8 (PMC6070490; doi:10.1038/s41598-018-29884-8)
Supplement: Supplementary file 1 — Supporting Information [file 41598_2018_29884_MOESM1_ESM.pdf]

FliS/flagellin/FliW heterotrimer couples type III secretion and flagellin homeostasis

Florian Altegoer[1], Sampriti Mukherjee[2], Wieland Steinchen[1], Patricia Bedrunka[1], Uwe Linne[1], Daniel B. Kearns[2], Gert Bange[1,*]

[1]LOEWE Center for Synthetic Microbiology & Dep. of Chemistry, Philipps University Marburg, Hans-Meerwein-Strasse 6, 35043 Marburg, Germany

[2]Department of Biology, Indiana University, 1001 East 3rd Street, Bloomington, IN 47405, USA

*Correspondence: gert.bange@synmikro.uni-marburg.de

Supporting information

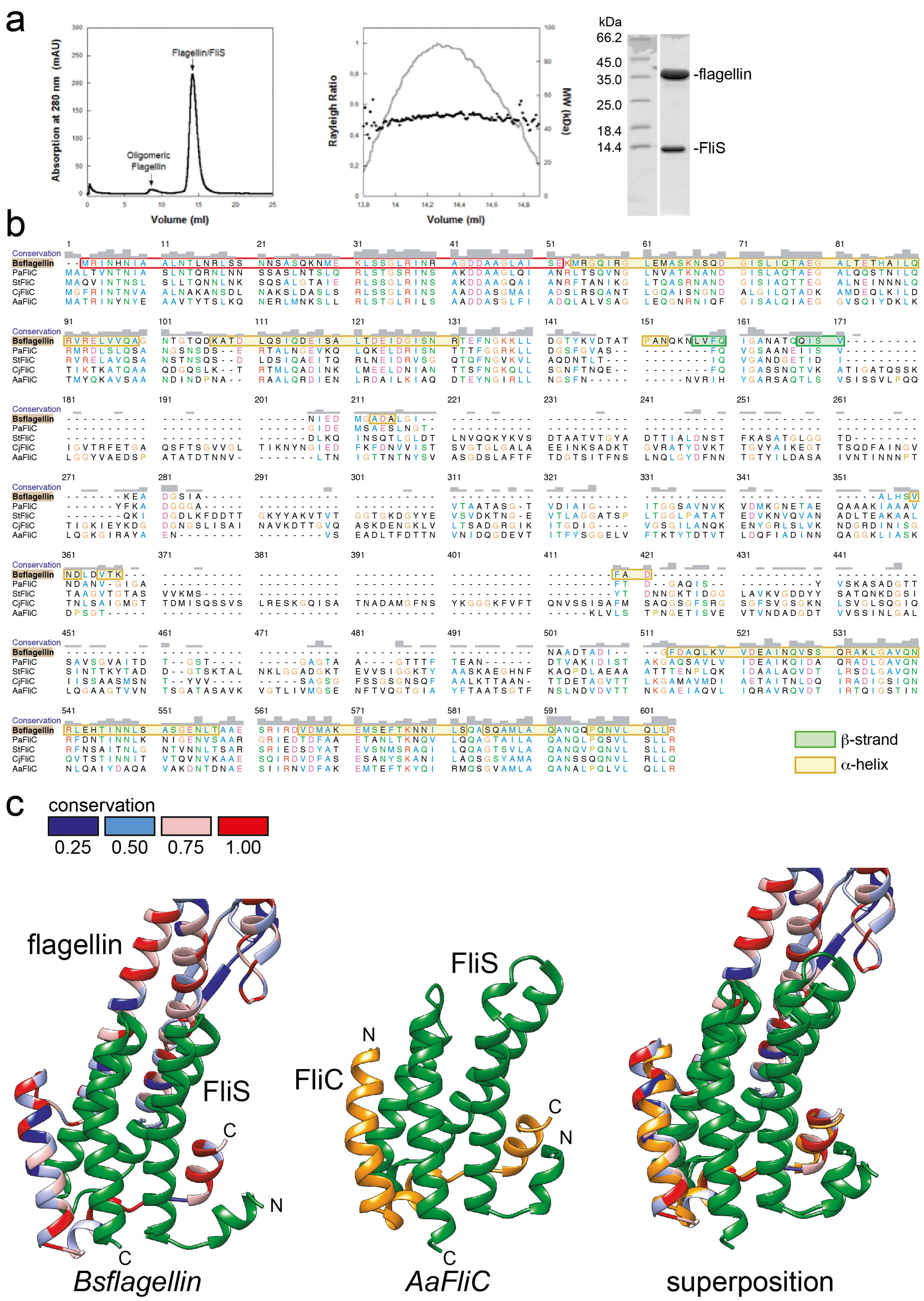

Figure S1. a. Flagellin and FliS form a heterodimer in solution. ***Left:*** Size-exclusion chromatography (SEC) chromatogram of flagellin/FliS (280 nm wavelength) ***middle:*** Analysis of the absolute molecular weight (MW) by static light scattering/refractive

index analysis. The Rayleigh ratio (left Y-scale) and the MW distribution (right Y-scale) of the main peak in the SEC is shown. The full-length Coomassie-stained SDS-PAGE is shown in Fig. S4B. The average MW of the Flagellin/FliS complex was 46.7 kDa ± 3 %. The calculated MW from the amino acid sequences of flagellin (33.5 kDa) and FliS (15.1 kDa) was 48.6 kDa. ***Right:*** Purity of the flagellin/FliS complex after SEC on Coomassie stained SDS-PAGE. **b.** Alignment of flagellin sequences from *B. subtilis* (Bs), *Pseudomonas aeruginosa* (Pa), *Salmonella typhimurium* (St), *Campylobacter jejuni* (Cj) and *Aquifex aeolicus* (Aa) residues color-coded according to chemical properties. Secondary structure elements refer to the *B. subtilis* crystal structure and are colored in yellow (α-helices) and green (β-strands). Red boxes indicate regions not resolved in the crystal structure. **c.** Structural alignment of *A. aeolicus* and *B. subtilis* FliS in the flagellin bound state. Flagellin from *B. subtilis* has been color-coded according to the amino acid conservation showing that the FliS-interface at flagellin is highly conserved. Figures have been generated in Chimera [1].

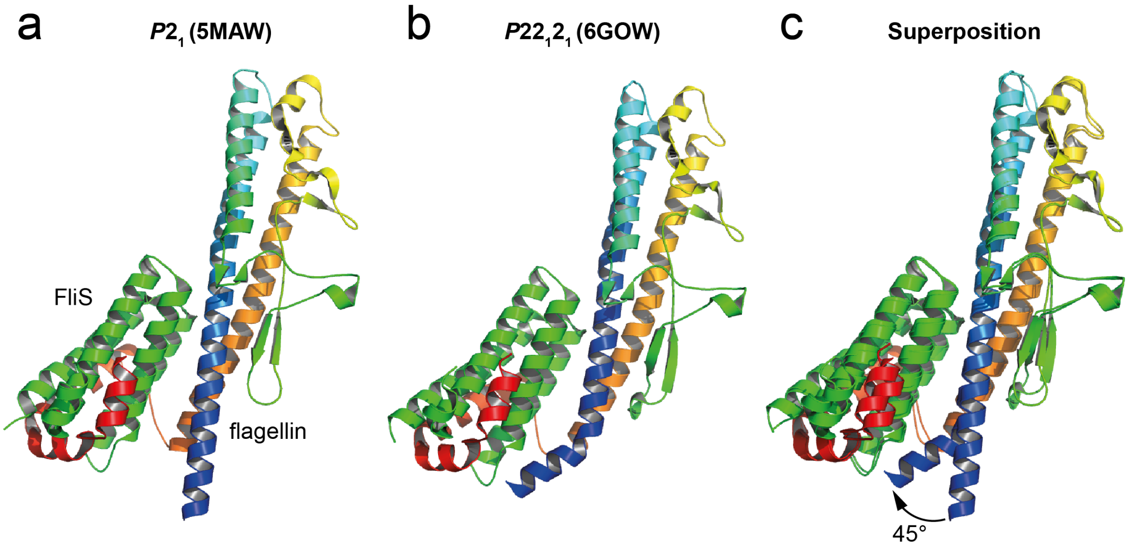

Figure S2. Flagellin/FliS crystallized in two different spacegroups. a. Crystal structure derived from primitive monoclinic crystals. **b.** Crystal structure derived from primitive orthorhombic crystals. **c.** Superposition of both crystal structures shows an r.m.s.d. over 329 c-α-atoms of 1.5. While the 45 N-terminal residues are not resolved in the crystal structure, residues 46 to 57 are kinked by 45° in the orthorhombic crystal structure. FliS is colored in green, flagellin is colored in rainbow from N- to C-terminus.

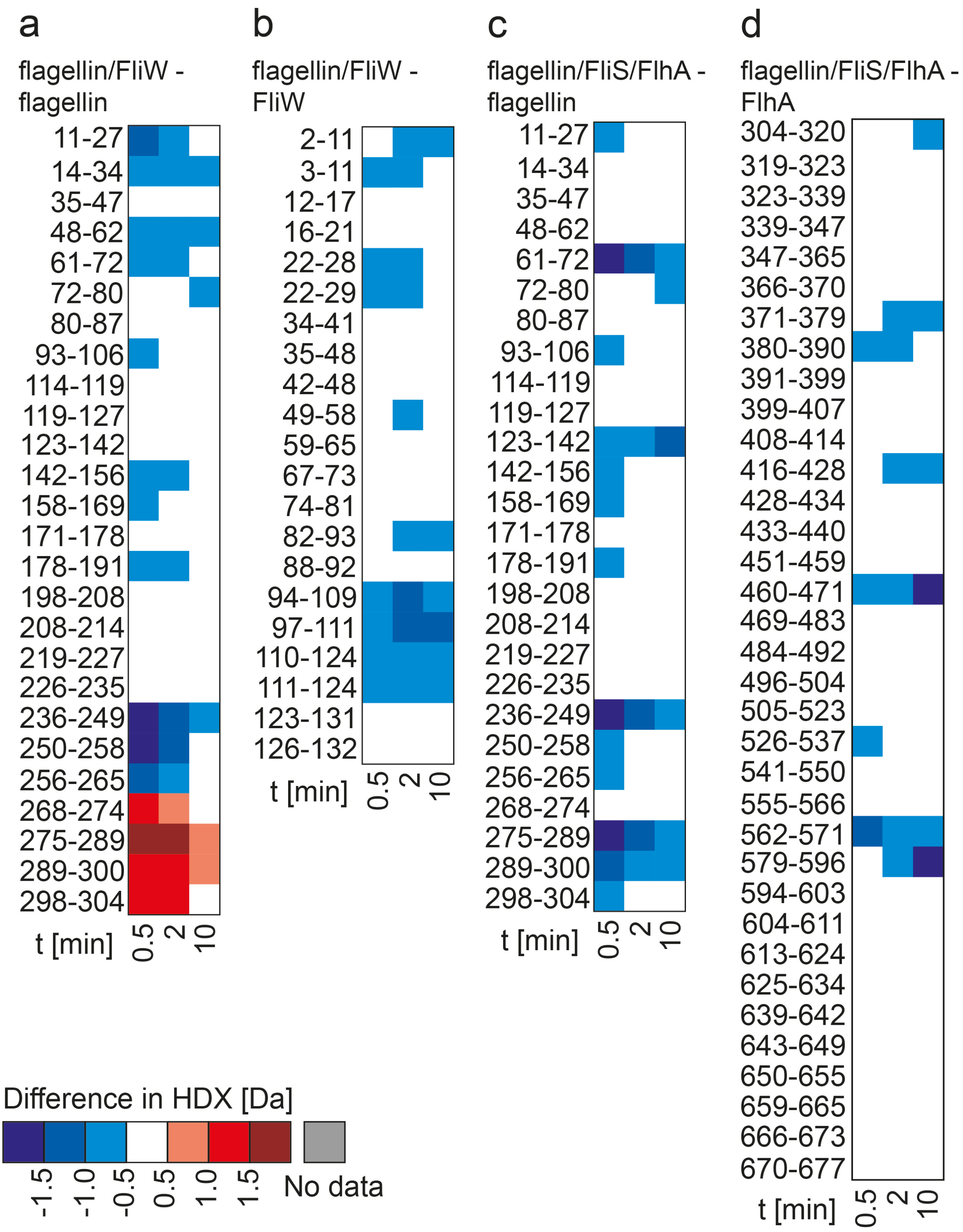

Figure S3. HDX-analysis of flagellin/FliW and flagellin/FliS/FlhA. a. Deuterium incorporation in the flagellin/FliW complex within flagellin compared to flagellin alone. **b.** Deuterium incorporation in the flagellin/FliW complex within FliW compared to FliW alone. **c.** Deuterium incorporation in the flagellin/FliS/FlhA complex within flagellin compared to flagellin/FliS alone. **d.** Deuterium incorporation in the flagellin/FliS/FlhA complex within FlhA-C compared to FlhA-C alone. The difference in HDX-labelling is

shown from blue (less exchange) to red (more exchange) in Dalton.

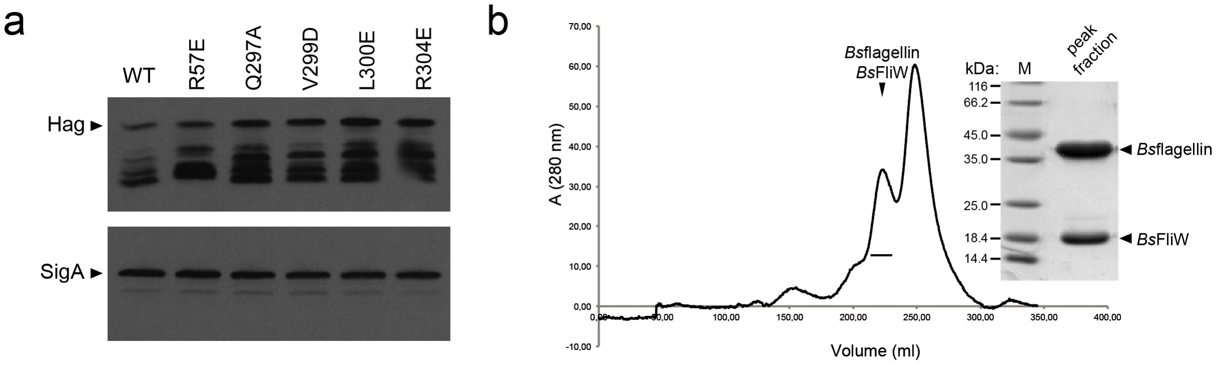

Figure S4. a. Proteolytic stability of Hag alleles. Western blot analysis of the Hag alleles indicated at the top of the panel probed with anti-Hag (top) and anti-SigA (bottom) respectively. Each allele was probed in a sensitized background mutated for the hook protein FlgE (to eliminate differences in Hag protein due to differential filament lengths) and CsrA (to eliminate homeostatic compensation due to enhanced cytoplasmic instability). The sizes of full length Hag and SigA (loading control) are indicated by carets. Although the enhanced abundance of Hag trapped in the cytoplasm resulted in proteolytic degradation in the sensitized background, the mutant alleles were not more unstable than wild type. The following strains were used to generate the panel from left to right: DK6192, DK6193, DK6194, DK6195, DK6196, and DK6197 **b. FliW prevents flagellin polymerization *in vitro*.** SEC chromatogram of the flagellin/FliW complex and a coomassie-stained SDS-PAGE of the peak fraction. The full-length Coomassie-stained SDS-PAGE is shown in Fig. S4E.

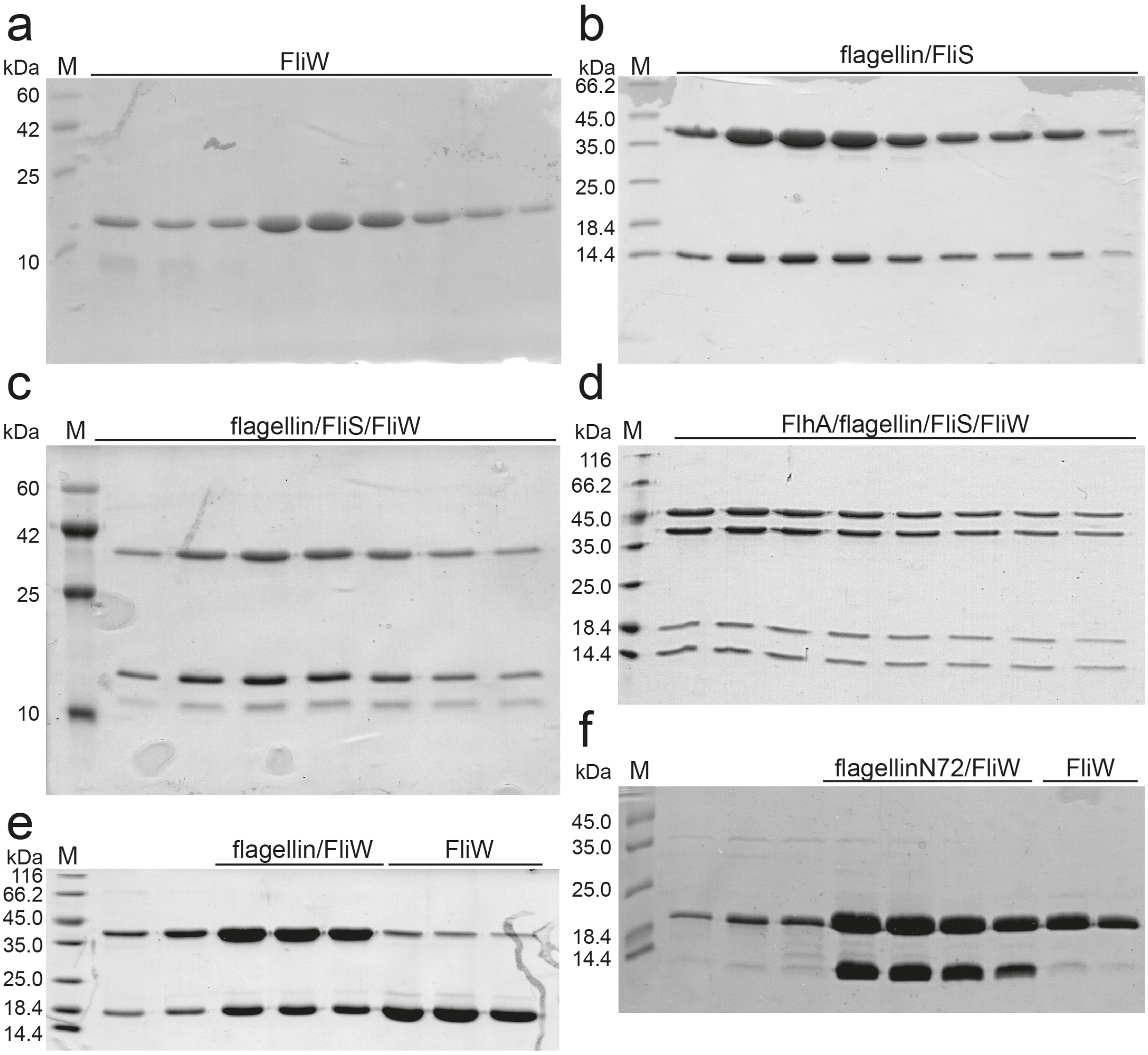

Figure S5. Coomassie stained full-length SDS-PAGEs of peak fractions from SEC. a. Peak fractions of a FliW SEC analysis. **b.** Peak fractions of a flagellin/FliS SEC analysis. **c.** Peak fractions of a flagellin/FliS/FliW SEC analysis. **d.** Peak fractions of a FlhA/flagellin/FliS/FliW SEC analysis. **e.** Peak fractions of a flagellin/FliW SEC analysis. **f.** Peak fractions of a flagellinN72/FliW SEC analysis.

Supplemental Tables

Table S1: Data collection and refinement statistics of the flagellin/FliS complex. Values in parenthesis refer to the highest resolution shell. For R_{free} calculation, 5 % of the total reflections from the working set were used.

		flagellin/FliS (5MAW)	flagellin/FliS (6GOW)
**Data collection**	Space group	$P2_1$	$P22_12_1$
	Cell dimensions		
	a, *b*, *c* (Å)	52.13	50.86
		62.35	89.95
		65.07	116.07
	α, β, γ (°)	90.00	90.00
		111.33	90.00
		90.00	90.00
	Energy (keV)	0.9724	0.987
	Resolution (Å)	48.57 - 1.5	48.77 – 2.1
		(1.55 - 1.5)	(2.175 – 2.1)
	R_{merge}	0.0439 (0.23)	0.073 (0.13)
	$I / \sigma I$	18.17 (5.40)	13.65 (7.17)
	Completeness (%)	0.99 (0.99)	0.99 (0.97)
	Redundancy	4.1 (4.2)	4.2 (4.0)
**Refinement**			
	Resolution (Å)	48.56 - 1.50	48.77 – 2.1
	No. reflections	61470	31628
	R_{work}/ R_{free}	17.3	17.5
		19.4	20.4
	No. atoms	3209	3150
	Protein	2846	2792
	Ligand	0	0
	Water	363	358
	R.m.s deviations		
	Bond lengths (Å)	0.009	0.003
	Bond angles (°)	0.97	0.60
	Ramachandran (%)		
	Preferred	98.33	97.78
	Allowed	1.4	2.22
	Outliers	0.27	0.00

Table S2: Strains

Strain	Genotype
3610	Wild type
DK980	$fliS^{\Delta 2\text{-}18}$ *amyE::*P_{hag}-hag^{T209C} *spec*
DK981	$fliS^{K33E}$ *amyE::*P_{hag}-hag^{T209C} *spec*
DK983	$fliS^{Y7A,\ Y10A}$ *amyE::*P_{hag}-hag^{T209C} *spec*
DK1118	*amyE::spec*
DK1123	*Δhag amyE::*P_{hag}-$hag^{T209C,\ V299D}$ *spec*
DK1124	*Δhag amyE::*P_{hag}-$hag^{T209C,\ L300E}$ *spec*

DK1125	Δhag amyE::P$_{hag}$-hag$^{T209C, R304E}$ spec
DK1163	Δhag amyE::P$_{hag}$-hag$^{R57E, T209C}$ spec
DK1164	Δhag amyE::P$_{hag}$-hag^{T209C,Q297A} spec
DK1208	amyE:: P$_{fla/che}$-flhA spec
DK1277	ΔflhA amyE::P$_{fla/che}$-flhA$^{\Delta 427DDLIE}$ spec lacA::P$_{hag}$-hag^{T209C} mls
DK1279	ΔflhA amyE::P$_{fla/che}$-flhA$^{\Delta 453DEADM}$ spec lacA::P$_{hag}$-hag^{T209C} mls
DK1302	ΔflhA lacA::P$_{hag}$-hag^{T209C} mls
DK1303	ΔflhA amyE::P$_{fla/che}$-flhA spec lacA::P$_{hag}$-hag^{T209C} mls
DK1337	ΔflhA amyE::P$_{fla/che}$-flhA$^{\Delta 445KWISE}$ spec lacA::P$_{hag}$-hag^{T209C} mls
DS1916	amyE::P$_{hag}$-hag^{T209C} spec
DS7812	ΔfliS amyE::P$_{hag}$-hag^{T209C} spec
DK6192	ΔflgE ΔcsrAhag amyE::P$_{hag}$-hag^{T209C} spec
DK6193	ΔflgE ΔcsrAhag amyE::P$_{hag}$-hag^{R57E,T209C} spec
DK6194	ΔflgE ΔcsrAhag amyE::P$_{hag}$-hag^{T209C,Q297A} spec
DK6195	ΔflgE ΔcsrAhag amyE::P$_{hag}$-hag^{T209C,V299D} spec
DK6196	ΔflgE ΔcsrAhag amyE::P$_{hag}$-hag^{T209C,L300E} spec
DK6197	ΔflgE ΔcsrAhag amyE::P$_{hag}$-hag^{T209C,R304E} spec

Table S3: Primers

Primer	Sequence
3177	aggagggatccctagttttatcgcggaaaataaacga
3180	ctcctgtcgacaccctcagctgtttggataagaga
3499	gtcgactctagaggatccccgagtacgtagaaacgattgcg
3500	cataagcgtcagctccccgggcatgtgtcatcctccaaattac
3501	taatttggatgacacatgcccggggagctgacgctt
3502	gtgaattcgagctcggtacccctttaaatactcacagaactcat
3503	gtcgactctagaggatcccccttatgtccggtcagcttg
3504	agcggcaagtcttataaattccaaacagccattatacagcata
3505	ctgtataatggctgtttggaatttataagacttgccgctcag
3506	gtgaattcgagctcggtaccccatacattgatgaatagacagc
3507	aatcagattttcatttttttccatatcatcattctcaatgg
3508	gagaatgatgatatggaagaaaaaaatgaaaatctgattaaagcg
3509	gtcgactctagaggatccccgtacgtagaaacgattgcgtt
3510	ttgctgtgcggctgttgctggattttggatcgccatgtg
3511	aatccagcaacagccgcacagcaaaattcagtgaatacgg
3512	gtgaattcgagctcggtacccctcatgagagggaaaagagaa
3557	ttttaaaattaacgtaataattgaaggtcgttttgcggctgttggtttg

3558	caaaacgtacttcaattattagagtaattttaaaaaagaccttggcg
3561	ttagaagccatttcaagaccctcgatttgtcctctcattttttcag
3562	aaaaaatgagaggacaaatcgagggtcttgaaatggcttctaaaa
3711	ctcaagcaaaccaacagccggcaaacgtacttcaattattacgttaa
3712	ctcaagcaaaccaacagccggcaaacgtacttcaattattacgttaa
3713	caaaccaacagccgcaaaacgaccttcaattattacgttaattttaaaa
3714	ttttaaaattaacgtaataattgaaggtcgttttgcggctgttggtttg
3715	aaccaacagccgcaaaacgtagagcaattattacgttaattttaaaaaag
3716	cttttttaaaattaacgtaataattgctctacgttttgcggctgttggtt
3728	gccgacacggtcagcaattcagaattcctgcagccctg
3729	aattgctgaccgtgtcggc
3730	tacgtaagccagaatttctgcttttcaccctcaatatccttgt
3731	gcagaaattctggcttacgta
3732	ctggtctgatcggatccatgatgctgaaaaatcacctagctc
3733	catggatccgatcagaccag
3771	gcgatgtcgcctactcctgaggggattgaaacagtggagcc
3772	aggctccactgtttcaatcccctcaggagtaggcgacatc
3773	ccttcattcggactgccggctgcggtaaaggatgaagccg
3774	gtcggcttcatcctttaccgcagccggcagtccgaatgaa
3775	tggattagcgaagcggtaaagcttggttatacagtagttgatc
3776	atcaactactgtataaccaagctttaccgcttcgctaatcc

Supplementary Material & Methods

Protein production and purification

The genes encoding for flagellin, FliS, FliW and FlhA proteins from *B. subtilis* were amplified from genomic DNA by polymerase chain reaction (PCR) and cloned into pET16b and pET24d vectors (Novagen) via the *Nco*I and *BamH*I restriction sites. Flagellin, FliW and FliS contained an N-terminal hexa-histidine tag, FlhA-C was tagged C-terminally. Proteins were produced in *E. coli* BL21 (DE3) (Novagen). After cell lysis by a Microfluidizer (M110-L, Microfluidics), cell debris was removed by high-speed centrifugation and proteins were purified by Ni-ion affinity- and size exclusion chromatography (SEC) as described recently [2]. The SEC buffer consisted of 20 mM HEPES-Na (pH 7.5), 200 mM NaCl, 20 mM KCl, and 20 mM $MgCl_2$.

Hydrogen-deuterium exchange mass-spectrometry

For HDX analysis of flagellin/FliS, FliW and FlhA-C, 200 pmol (4 µl of 50 µM solution) of protein were incubated without or in the presence of binding partners for 5 min at 37 °C prior to H/D exchange. Binding partners were added to the respective protein in equimolar concentrations. The mixtures were diluted 10-fold in D_2O-containing SEC buffer to start the H/D exchange and incubated at 37 °C. The reactions were stopped after different incubation times (i.e. 30, 120, 600 sec) through addition of an equal volume of ice-cold quench buffer and directly injected into an ACQUITY UPLC M-class system with HDX technology (Waters)[3,4].

All proteins were digested online using an Enzymate BEH Pepsin column 2.1 x 30 mm (Waters) at a flow rate of 100 µl/min ddH_2O + 0.1 % (v/v) formic acid at 11 °C and the resulting peptic peptides trapped for 3 min using an AQUITY UPLC BEH C18 1.7 µm 2.1 x 5 mm VanGuard Pre-column (Waters) kept at 0.5 °C [5]. Thereafter, the trap column was placed in line with an ACQUITY UPLC BEH C18 1.7 µm 1.0 x 100 mm column (Waters) and the peptides eluted at 0.5 °C using a gradient of water + 0.1 % formic acid (HDX buffer A) and acetonitrile + 0.1 % formic acid (HDX buffer B) at 40 µl/min flow rate: 5% B (0 min), 5-35% B (0-7 min), 35-85% B (7-8 min), 85% B (8-10 min), 85-95% B (10-10.1 min), 95% B (10.1-11 min), 95-5% B (11-11.1 min), 5% B (11.1-16 min). Mass spectra were acquired in positive ion mode using a SYNAPT G2-Si mass spectrometer equipped with an electrospray ionization source (Waters). Deuterated peptides were detected in High Definition MS (HDMS, [6]) mode including ion mobility separation (IMS). Lock mass spectra were obtained every 30-45 s using [Glu1]-Fibrinopeptide B standard (Waters). Undeuterated peptides of all proteins were obtained similar as described above by 10-fold dilution in H_2O-containing SEC buffer and detected in Enhanced High Definition MS ($HDMS^E$) mode including IMS of precursor ions within the gas phase and alternating high and low energies applied to the transfer cell (Waters). All measurements were performed in triplicates. Blank runs were performed between each sample to avoid peptide carry-over.

Analysis of HDX data was aided by the softwares Protein LynX Global Server (PLGS) and DynamX 3.0 (both Waters). Identification of undeuterated peptides was performed using PLGS with custom-created databases and the setting ‘no enzyme’. Only peptides identified in at least two replicates of each nucleotide-bound state were used for assignment of deuterium incorporation in DynamX 3.0. Thresholds of 0.5 min and 25 ppm for retention time and *m/z* values, respectively, were applied for assignment of

the deuterated peptides to their undeuterated counterparts. Deuterium incorporation into each peptide was calculated by subtracting the centroid of the isotope distribution of the undeuterated from the deuterated peptides. Relative deuteration was calculated as the quotient between absolute deuteration and the number of backbone amide hydrogens of the peptide [6].

Strain construction

Complementation constructs. To generate the *amyE::*P_{hag} –*hag*^{R57E,T209C} *spec* site-directed mutant complementation construct, PCR products were amplified from the strain DS1916 as template using primer pairs 3177/3561 and 3562/3180 such that the primers 3561 and 3562 were complementary in sequence and contained the R57E mutation. The amplicons were ligated using Gibson assembly. The *amyE::*P_{hag} –*hag*^{Q297A,T209C} *spec* site-directed mutant complementation construct was generated similarly using primer pairs 3177/3712 and 3711/3180. The *amyE::*P_{hag} –*hag*^{V299D,T209C} *spec* site-directed mutant complementation construct was generated similarly using primer pairs 3177/3714 and 3713/3180. The *amyE::*P_{hag} –*hag*^{L300E,T209C} *spec* site-directed mutant complementation construct was generated similarly using primer pairs 3177/3716 and 3715/3180. The *amyE::*P_{hag}–*hag*^{R304E,T209C} *spec* site-directed mutant complementation construct was generated similarly using primer pairs 3177/3557 and 3558/3180.

To generate the *amyE::*$P_{fla/che}$*-flhA* spec complementation construct, PCR products containing the $P_{fla/che}$ promoter and the *flhA* coding region were amplified from *B. subtilis* 3610 chromosomal DNA using the primer pairs 3729/3730 and 3731/3732, DNA fragment containing a spectinomycin resistance cassette and *amyE* region were amplified from the strain DK1118 as template using primers 3177/3728 and 3733/3180, and the PCR products were ligated using Gibson assembly (Gibson et al 2009).

To generate the *amyE::*$P_{fla/che}$*-flhA*$^{\Delta 427DDLIE}$ *spec* site-directed mutant complementation construct, PCR products were amplified from the strain DK1208 as template using primer pairs 3177/3772 and 3771/3180 such that the primers 3771 and 3772 were complementary in sequence and contained the Δ427DDLIE deletion mutation. The amplicons were ligated using Gibson assembly. The *amyE::*$P_{fla/che}$*-flhA*$^{\Delta 445KWISE}$ *spec* site-directed mutant complementation construct was generated similarly using primer pairs 3177/3774 and 3773/3180. The *amyE::*$P_{fla/che}$*-flhA*$^{\Delta 453DEADM}$ *spec* site-directed

mutant complementation construct was generated similarly using primer pairs 3177/3776 and 3775/3180.

Allelic replacement. The *fliS*$^{\Delta2\text{-}18}$ allele was generated using isothermal "Gibson" assembly (Gibson et al. 2009). A fragment containing the 5'-end of *fliS* and approximately 1000 bp upstream of the gene was PCR amplified using the primer pair 3499/3500 and a fragment containing the 3'-part of *fliS* and approximately 650 bp downstream of the gene was PCR amplified using the primer pair 3501/3502. Primers 3500 and 3501 are reverse complements and were used to delete codons 2-18 of *fliS*. Primers 3499 and 3502 contain a short sequence (~20 bp) with homology to pMiniMadII linearized with SmaI. The three DNA fragments were combined at equimolar amounts and assembled to generate the *fliS*$^{\Delta2\text{-}18}$ allelic replacement construct.

The *fliS*^{K33E} allelic replacement construct was generated similarly using primer pairs 3503/3504 and 3505/3506. The *fliS*$^{Y7A,\ Y10A}$ allelic replacement construct was generated similarly using primer pairs 3509/3510 and 3511/3512.

Western blotting. *B. subtilis* strains were grown in LB to OD_{600} ~1.0, 1 ml was harvested by centrifugation, and resuspended to 10 OD_{600} in Lysis buffer (20 mM Tris pH 7.0, 10 mM EDTA, 1 mg/ml lysozyme, 10 μg/ml DNAse I, 100 μg/ml RNAse I, 1 mM PMSF) and incubated 30 minutes at 37°C. 10 μl of lysate was mixed with 2 μl 6x SDS loading dye. Samples were separated by 12% Sodium dodecyl sulfate-polyacrylamide gel electrophoresis (SDS-PAGE). The proteins were electroblotted onto nitrocellulose and developed with a 1:40,000 dilution of primary antibody (either anti-Hag or anti-SigA) and a 1:10,000 dilution secondary antibody (horseradish peroxidase-conjugated goat anti-rabbit immunoglobulin G). Immunoblot was developed using the Immun-Star HRP developer kit (Bio-Rad).

Supplementary References

1. Pettersen, E. F. *et al.* UCSF Chimera: A visualization system for exploratory research and analysis. *J. Comput. Chem.* **25,** 1605–1612 (2004).
2. Schuhmacher, J. S. S. *et al.* MinD-like ATPase FlhG effects location and number of bacterial flagella during C-ring assembly. *Proc. Natl. Acad. Sci.* **112,** 201419388 (2015).

3. Schäper, S. *et al.* AraC-like transcriptional activator CuxR binds c-di-GMP by a PilZ-like mechanism to regulate extracellular polysaccharide production. **114,** E4822–E4831 (2017).
4. Steinchen, W. *et al.* Catalytic mechanism and allosteric regulation of an oligomeric (p)ppGpp synthetase by an alarmone. *Proc. Natl. Acad. Sci. U. S. A.* **112,** 13348–53 (2015).
5. Wales, T. E., Fadgen, K. E., Gerhardt, G. C. & Engen, J. R. High-Speed and High-Resolution UPLC Separation at Zero Degrees Celsius. *Anal. Chem.* **80,** 6815–6820 (2008).
6. Li, G.-Z. *et al.* Database searching and accounting of multiplexed precursor and product ion spectra from the data independent analysis of simple and complex peptide mixtures. *Proteomics* **9,** 1696–1719 (2009).
